# TCNQ-based organic cocrystal integrated red emission and n-type charge transport

**DOI:** 10.1007/s12200-022-00022-7

**Published:** 2022-05-09

**Authors:** Mengjia Jiang, Shuyu Li, Chun Zhen, Lingsong Wang, Fei Li, Yihan Zhang, Weibing Dong, Xiaotao Zhang, Wenping Hu

**Affiliations:** 1 Tianjin Key Laboratory of Molecular Optoelectronic Sciences, Department of Chemistry, School of Science, Tianjin University, Tianjin, 300072 China; 2 Institute of Molecular Aggregation Science, Tianjin University, Tianjin, 300072 China; 3 Key Laboratory of Resource Chemistry and Eco-Environmental Protection in Qinghai-Tibet Plateau, School of Chemistry and Chemical Engineering, Qinghai Minzu University, Xining, 810007 China; 4 Joint School of National University of Singapore and Tianjin University, International Campus of Tianjin University, Fuzhou, 350207 China

**Keywords:** Organic cocrystal, Charge transfer (CT), Integrated optoelectronic properties, Red emission, n-type charge transport

## Abstract

Simultaneously realizing the optical and electrical properties of organic materials is always challenging. Herein, a convenient and promising strategy for designing organic materials with integrated optoelectronic properties based on cocrystal engineering has been put forward. By selecting the fluorene (Flu) and the 7,7′,8,8′-tetracyanoquinodimethane (TCNQ) as functional constituents, the Flu-TCNQ cocrystal prepared shows deep red emission at 702 nm, which is comparable to the commercialized red quantum dot. The highest electron mobility of organic field-effect transistor (OFET) based on Flu-TCNQ is 0.32 cm^2^ V^−1^ s^−1^. Spectroscopic analysis indicates that the intermolecular driving force contributing to the co-assembly of Flu-TCNQ is mainly charge transfer (CT) interaction, which leads to its different optoelectronic properties from constituents.

**Graphical Abstract:**

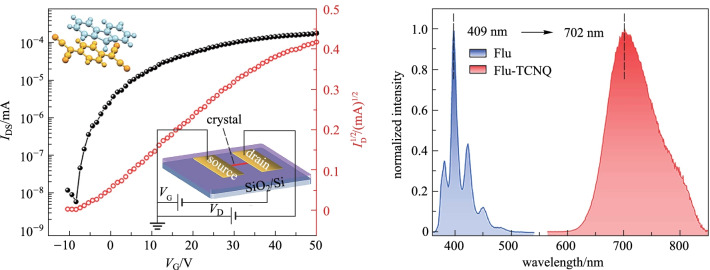

**Supplementary Information:**

The online version contains supplementary material available at 10.1007/s12200-022-00022-7.

## Introduction

Organic semiconductor materials with characteristics of light weight, flexibility, easy-preparation, large-area solution processing, and low cost have been demonstrated in great applications. Examples are organic field-effect transistor (OFET), organic light-emitting diode (OLED), organic phototransistor (OPT), and organic photovoltaic (OPV) devices [1–6]. Besides single performance optoelectronic materials, organic materials with integrated optoelectronic characteristics are gaining more attention which can serve as candidates for multifunctional optoelectronic devices and integrated circuit fabrication. Examples are the organic light-emitting transistor (OLET) (a device that integrates the functions of OFET and OLED), and pumped organic lasers (EPOLs) [7–10]. In recent years, some organic optoelectronic materials with n/p type charge-carrier mobility and light emission properties have been reported [11–13]. However, most of them exhibit a blue or green emission, and few materials exhibit red emission. In addition, these optoelectronic materials mainly display p-type charge transport behavior [14–16]. Designing and constructing materials with red emission and n-type charge transport is of research significance, but requires a lot of effort. To date, some researchers designed and synthesized materials that combined red emission and n-type charge transfer (CT). Takahiro Kono et al. utilized the efficient fluorophore and small bandgap of dithienylbenzothiadiazole derivative by introducing trifluoromethylphenyl groups, developed an optoelectronic material with red fluorescence and n-type mobility. The benzoselenadiazole and quinoxaline derivatives also exhibit similar optoelectronic properties [17]. Sangyoon Oh et al. investigated the red photoluminescence of an n-type dicyanodistyrylbenzene (DSC-type) derivatives for the first time, and reported the integrated optoelectronic properties of ((2E,2′E)-3,3′-(2,5-*bis*(hexyloxy)-1,4-phenylene) *bis*(2-(5-(4-(trifluoromethyl) phenyl) thiophen-2-yl) acrylonitrile) (Hex-4-TFPTA). They then fabricated the OLET by using the Hex-4-TFPTA as the active layer [18].

Cocrystal is a more ingenious strategy to achieve the integrated optoelectronic properties by noncovalent assembling two or more constituents through intermolecular interactions such as π–π interactions, CT interactions, halogen bonds, and hydrogen bonds [19–21]. The individual constituents can exhibit their inherent properties, and some novel properties also emerge owing to the cooperative effect [22, 23]. Therefore, co-crystallization engenders a promising way for designing and synthesizing the integrated optoelectronic materials with luminescence and charge transport properties, by adjusting donors and acceptors. Yet, many researchers currently focus on constructing materials with the single property of red emission [24, 25] or charge transport [26–28]. Only the Soo Young Park group, designed a series of cocrystals with red emission and charge transport properties through co-assemble molecules that have a 1,4-distyrylbenzene skeleton [29, 30]. The advantages of cocrystals in constructing integrated optoelectronic materials have not been fully utilized.

Herein, the fluorene (Flu) was selected as the donor due to its good luminescence, extended π-conjugated plans, and rich electron properties, which should be an ideal constituent for assembling cocrystals with optoelectronic properties. According to previous reports, the Flu has been used as the luminescence unit to be introduced to special electron-withdrawing group and realized the red emission [31]. As well, 7,7′,8,8′-tetracyanoquinodimethane (TCNQ) was chosen as the electrical building block, a typical n-type semiconductor that can provide a strong electron-withdrawing capacity. Both compositions were easily obtained, avoiding the tedious synthetic routes. The strong CT interaction between donor–acceptor (D–A) pairs is responsible for the co-crystallization. As expected, the binary Flu-TCNQ cocrystal combines the optical and electrical properties of two constituents. Devices fabricated by Flu-TCNQ cocrystal exhibit integrated optoelectronics of deep red luminescence and high n-type charge transport.

## Experimental and details

### Materials and preparations

Fluorene (Flu, CAS registry no. 86-73-7, 99%) was purchased from Shanghai Aladdin Co., Ltd. (China), and 7,7,8,8,-Tetracyanoquinodimethane (TCNQ, CAS registry no. 1518-16-7, 98%) was purchased from Shanghai TCI Co., Ltd. (China). The dichloromethane solvent and acetonitrile solvents with high performance liquid chromatography (HPLC) grade, were purchased from Shanghai Maclin Co., Ltd. (China).

**Fig. 1:**
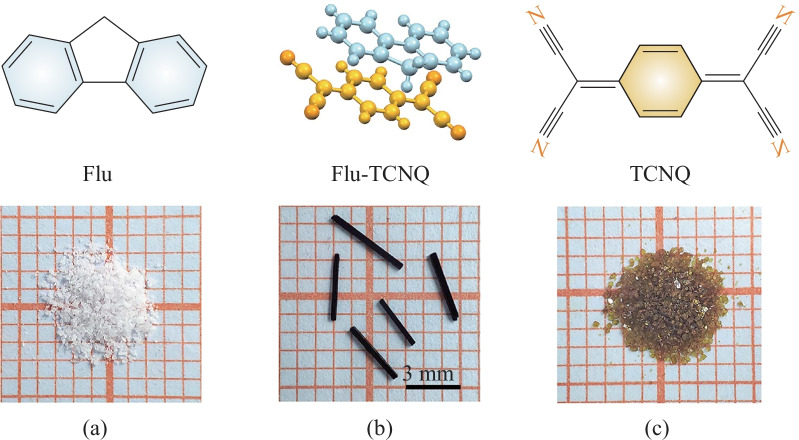
Optical images of **a** Flu powder, **b** Flu-TCNQ crystal, and **c** TCNQ powder with the chemical structures

To grow Flu-TCNQ cocrystals for single-crystal X-ray diffraction characterization, 0.06 mmol Flu and 0.06 mmol TCNQ were dissolved in 10 mL dichloromethane solution at room temperature. The large rod-like Flu-TCNQ cocrystals with good quality were obtained after slow evaporation for five days, and their dark red color was distinct from the pure components (Fig. 1a−c). The Flu-TCNQ microwires for OFET fabrication were prepared by the drop-casting method [32]. In a typical experiment, the Flu and TCNQ with a 1:1 ratio was mixed in an acetonitrile solvent. Then the 5−8 μL of mixture solution was immediately dropped onto the modified Si/SiO_2_ substrate, and cocrystal microwires were obtained when the solvent in the droplet completely evaporated.

### Device fabrication

To remove impurities, the bare Si wafer (10 mm × 10 mm) was successively washed with pure water, acetone solution, and isopropanol solution in an ultrasonic cleaner for 10 min, respectively. Then the substrate was treated with oxygen plasma for 10 min, followed by the *n*-octadecyl trichlorosilane (OTS) modification via a vapor deposition method. Subsequently, the solution of Flu-TCNQ was dropped onto the prepared substrate to grow microcrystals. The bottom-gate top-contact OFET based on microcrystal was fabricated with stamping Au electrodes by the probe [32, 33]. The measurements of OFET characteristics were performed on Keithley 4200 SCS (USA) and the 6150-probe station, under an ambient condition. The mobility was calculated through

1 $$I_{{{\text{DS}}}} = \, \left( {W/\left( {{2}L} \right)} \right)C_{{\text{i}}} \mu (V_{{\text{G}}} - V_{{{\text{TH}}}} )^{{2}} ,$$

where *L* and *W* are the length and width of the channel, respectively; *V*_G_ and *V*_TH_ are the gate voltage and threshold voltage, respectively; *C*_i_ is the capacitance per unit area of the insulating layer, *μ* is the mobility, *I*_DS_ is the drain current.

### General method

Single-crystal structure data of Flu-TCNQ were collected on a Rigaku Supernova X-ray diffractometer (Japan) with a Cu target at 293 K (40 kV, 30 mA). Powder X-ray diffraction (PXRD) experiment was carried out by a Rigaku SmartLab (Japan) equipment with a Cu target (*λ* = 1.542 Å, 40 kV, 200 mA). Thermogravimetric analysis (TGA) was performed on a Mettler Toledo instrument (Switzerland), and the heating temperature was set to rise from 25 °C to 500 °C. The solid-state ultraviolet–visible absorption (UV) measurements were conducted on Shimadzu UV-3600 Plus spectrophotometer (Japan) with an integrating sphere in a diffuse reflection mode. Fourier transform infrared (FTIR) spectrum was obtained on an FTIR spectrometer of Bruker Vertex 70 (Germany). The Raman spectra were acquired on a Renishaw Raman spectrometer (UK) with a 785 nm laser. The electron spin resonance (ESR) test was carried out on a Bruker EMXplus instrument (Switzerland) at room temperature. Photoluminescence (PL) spectroscopies were taken on an Edinburgh FLS1000 instrument (UK). The photoluminescence lifetime (PL lifetime) was measured using a picosecond pulse laser (EPL-340) on the Edinburgh FLS1000. The calculation of average PL lifetime (*τ*) accords to

2 $$\tau = \, \left( {B_{{1}} \tau_{{1}}^{{2}} + B_{{2}} \tau_{{2}}^{{2}} } \right)/\left( {B_{{1}} \tau_{{1}} + B_{{2}} \tau_{{2}} } \right),$$

where *τ*_1_ and *τ*_2_ are the double-exponential values of fitted lifetime, *B*_1_ (2234.0684) and *B*_2_ (947.5385) are the corresponding proportion, respectively. The photoluminescence quantum yield (PLQY) was obtained by using an integrating sphere of the Edinburgh FLS1000 spectrometer (UK).

## Results and discussion

### Cocrystal structure characterization

**Fig. 2:**
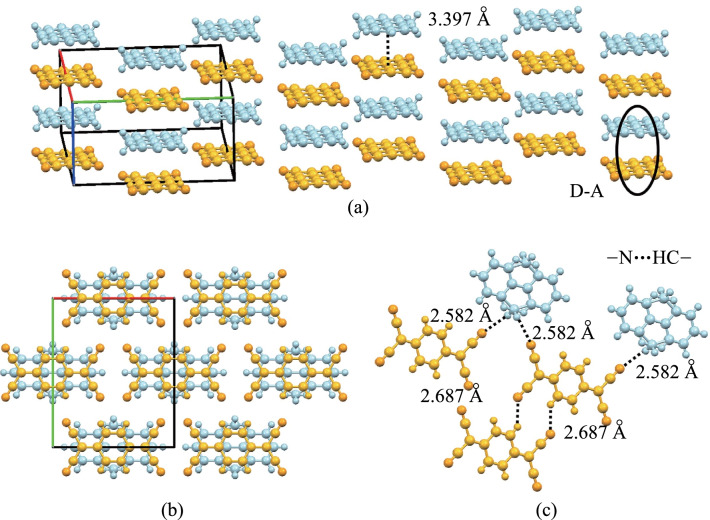
**a**, **b** Mixed packing structure of Flu-TCNQ (CCDC 2156827). **c** Intermolecular interactions in donor and acceptor molecules of Flu-TCNQ

To confirm the packing mode and the intermolecular interactions of Flu-TCNQ cocrystal, the single-crystal diffraction was carried out (CCDC 2156827). The crystallographic data denote that the Flu-TCNQ is monoclinic and belongs to the C2/m space group with the lattice parameters of *a* = 11.0097(6) Å, *b* = 13.1035(6) Å, *c* = 6.7937(3) Å, *α* = 90°, *β* = 103.545(5)°, *γ* = 90°, *V* = 952.84(8) Å^3^ (Additional file 1: Table S1). Figure 2a, b present the mixed packing structure of Flu-TCNQ, wherein the Flu molecule stacks over the center of the TCNQ molecule [34]. In a –D–A–D–A–… column, the Flu and TCNQ pack with a face-to-face mode along the *c* axis, and the D–A distance along the π–π direction is 3.397 Å (Additional file 1: Fig. S1), ensuring the strong intermolecular interaction in Flu-TCNQ [35]. Note that the Flu molecule appears disordered in the Flu-TCNQ cocrystal, because the five-membered ring of Flu occupies two symmetrically positions with the equivalent probability of 50% [36]. Besides, there are multiple C–H…N intermolecular interactions in Flu-TCNQ molecules, as illustrated in Fig. 2c. The distance of C–H…N between TCNQ molecules is 2.687 Å, while that of Flu and TCNQ molecules is 2.582 Å. This hydrogen-bond network not only makes the molecule structure more compact but also promotes the Flu-TCNQ to grow with a rod-like morphology. The packing potential energy was calculated through Mercury software, the energy between the adjacent donor and acceptor molecule is strongest (− 56.0 kJ/mol) (Additional file 1: Fig. S2), revealing the intermolecular driving force for the self-assembly of Flu-TCNQ.

**Fig. 3:**
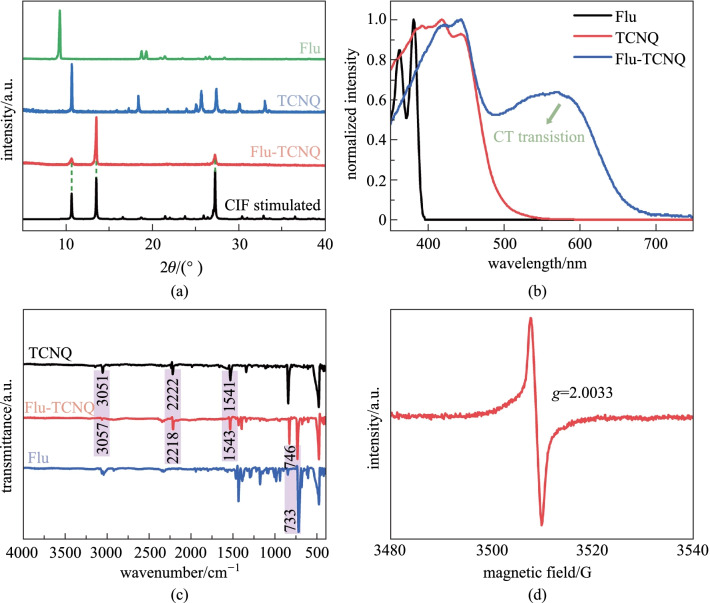
**a** XRD patterns, **b** UV–Vis absorption spectra, and **c** FTIR spectra of Flu, TCNQ, Flu-TCNQ. **d** ESR spectra of Flu-TCNQ

The powder X-ray diffractometer (PXRD) measurements were conducted for a deeper understanding of the cocrystal structure. In Fig. 3a, the PXRD patterns of Flu-TCNQ and the source powders are distinctly different, indicating the Flu-TCNQ is not the simple mixture of two compounds [37]. Additionally, the new finger peaks of cocrystal with sharp shapes and strong intensities suggest the better crystallinity of the cocrystal. These results of Flu-TCNQ measured by PXRD are in accordance with the simulated data of the CIF file. The thermogravimetric analysis (TGA) was used to evaluate the thermal stability of Flu-TCNQ (Additional file 1: Fig. S3). The smooth curves show that the sublimation points of the pristine Flu and TCNQ are 149 °C and 241 °C, respectively. In the formed cocrystal, the corresponding gradients exhibit higher sublimation points, 199 °C for the Flu component and 251 °C for the TCNQ component. It can be seen that the Flu molecules were completely lost when the temperature was raised to 245 °C. The 54.05% mass loss is consistent with the actual weighting mass ratio of the D–A molecules in Flu-TCNQ. The above analyses indicate that the Flu-TCNQ becomes more stable under co-assembly [38].

### Charge transfer nature and spectra measurements

In consideration of the critical effect of the intermolecular interaction on the crystal properties, a series of spectroscopies were conducted to determine the CT nature and unveil the physicochemical properties of Flu-TCNQ. We measured and analyzed the UV–vis absorption spectra of the as-prepared cocrystal and two constituents (Fig. 3b). It can be observed that the absorption peak of the Flu-TCNQ exhibits significant red-shift and is located at 574 nm, while the absorption peaks of the two pristine components both appear before 500 nm. The absorption band in the region from 510 to 630 nm is assigned as the CT band [39]. In the Fourier transform infrared (FTIR) spectra (Fig. 3c), the peaks of Flu and TCNQ can be recognized from those of Flu-TCNQ, demonstrating the existence of Flu and TCNQ in the as-obtained cocrystal. Specifically, the bands at 3051 cm^−1^ (C–H stretching), 2222 cm^−1^ (C≡N stretching), 1541 cm^−1^ (C=C stretching) in TCNQ are shifted to 3057, 2218, and 1543 cm^−1^ in Flu-TCNQ, respectively, implying the enhanced electron cloud density of the benzene rings in TCNQ molecules [40]. And the band at 733 cm^−1^ in Flu (C–H out-of-plane bending) is shifted to a high wave-number with respect to the 746 cm^−1^ in Flu-TCNQ. These slight movements of peaks are owing to the intermolecular force derived from CT interaction between donors and acceptors. Raman spectra were also recorded to confirm the composition of the cocrystal. As depicted in Additional file 1: Fig. S4, the peaks of Flu-TCNQ are almost the combination of these two constituents. Some small shifts are attributed to the polarization and delocalization of electrons between D–A molecules, which facilitate the formation of the CT system in Flu-TCNQ [41]. At the same time, the electron spin resonance spectra (ESR) of Flu-TCNQ was obtained at room temperature (Fig. 3d) The sharp signal that corresponds with a g factor of 2.0033 implies there are unpaired electrons in the ground state, further proving the CT process in the cocrystal [42].

### Optical properties

**Fig. 4:**
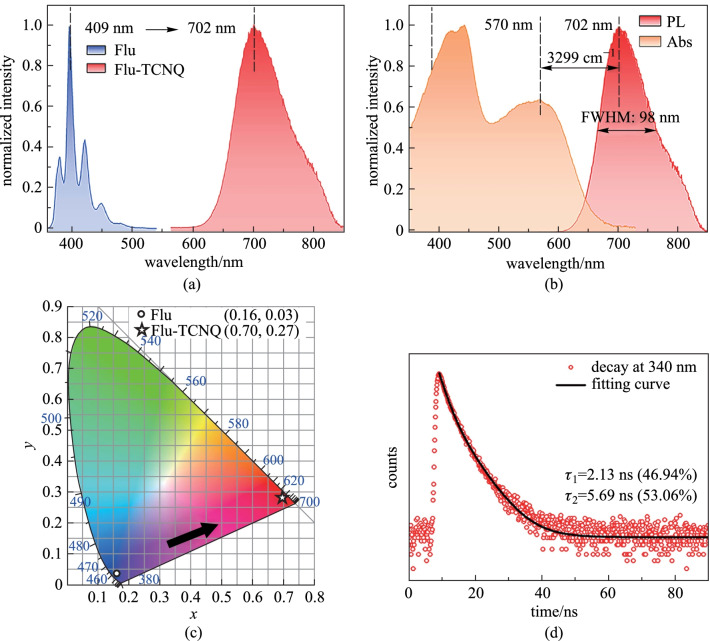
**a** PL spectra of Flu and Flu-TCNQ. **b** Normalized UV–Vis absorption spectrum and PL spectrum of Flu-TCNQ. **c** CIE 1931 chromaticity diagram and **d** PL decay curve of Flu and Flu-TCNQ

Driven by the strong intramolecular force, the Flu-TCNQ cocrystal exhibits unique integrated optoelectronic properties. According to the PL spectra, the photophysical properties of Flu-TCNQ were comprehensively researched. Remarkably, compared with the main emission peak of fluorene at 409 nm, the emission peak of Flu-TCNQ is bathochromic shifted to 702 nm (Fig. 4a), implying the CT interaction in the cocrystal, which induces the electron delocalization from Flu to TCNQ [43]. At the same time, the large Stokes shift is 3299 cm^−1^ that is usually less than 3000 cm^−1^ in a single component, and the full width at half-maximum (FWHM) is broadened to 98 nm (Fig. 4b). Besides, the calculated CIE coordinate displays an evident change from Flu (0.16, 0.03) to Flu-TCNQ (0.70, 0.27). (Fig. 4c), demonstrating the successful modulation of luminescent color from blue to the red region by co-assembly. The red color purity is comparable to the (0.71, 0.29) of commercialized red quantum dot [44]. The fluorescence microscope image of Flu-TCNQ microwires with bright red emission is shown in Additional file 1: Fig. S5. Moreover, the fitting PL decay curve with a double-exponential model (fitting constant *X*^2^ = 1.1493) was given to better understand the fluorescence properties of the solid-state cocrystal (Fig. 4d). As a result, the average PL lifetime value (*τ*) is 3.19 ns calculated from the fixed value of *τ*_1_ = 2.13 ns (46.94%) and *τ*_2_ = 5.69 ns (53.06%) [45]. Meanwhile, the solid-state photoluminescence quantum yield (PLQY, ФF) presents 1.44%.

### Electric properties and devices

**Fig. 5:**
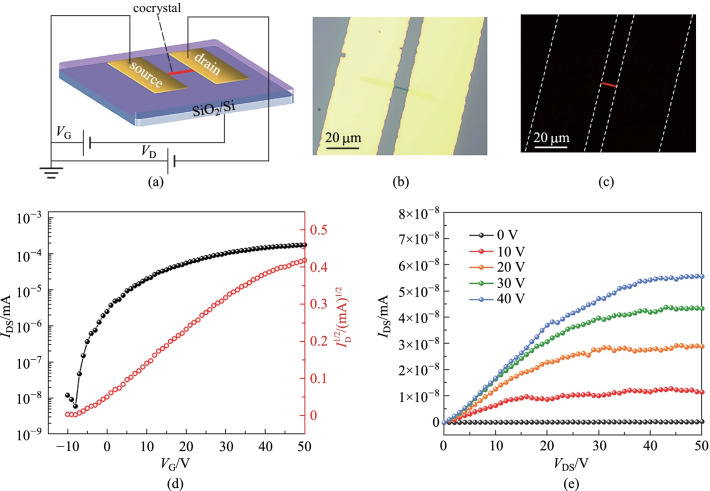
**a** Schematic diagram of bottom-gate and top-contact OFET device based on a single Flu-TCNQ crystal. **b** Optical microscope image (under visible light) and **c** fluorescence microscope image (under UV) of a typical Flu-TCNQ based OFET. **d** Transfer characteristic of the Flu-TCNQ based OFET at *V*_DS_ = 50 V (*L* = 9.93 μm, *W* = 0.44 μm). **e** Output characteristic of the Flu-TCNQ based OFET scanning from *V*_DS_ = 0 to 50 V

To investigate the electrical properties of Flu-TCNQ, we fabricated the bottom-gate top-contact OFETs by depositing the microcrystals on Si/SiO_2_ substrate (Fig. 5a). Figure 5b, c display the optical and fluorescence microscope images of a typical device based on an individual cocrystal microwire. The transfer and output characteristics of the OFET measured in a hole-enhancement mode are shown in Fig. 5d, e. From the transfer curve, the current between the source and drain electrodes appears at a low gate volt (*V*_G_), and then it increases with the increasing positive *V*_G_, showing a typical n-type charge transport behavior [46]. The highest electron mobility of the OFET extracted from the saturation region can reach 0.32 cm^2^ V^−1^ s^−1^ (*V*_DS_ = 50 V), and the on/off ratio is 10^5^. Besides, the average electron mobility calculated from 10 OFET devices is 0.25 cm^2^ V^−1^ s^−1^. Notably, there may be no direct electron transport channel between the TCNQ molecules due to the mixed packing mode of the Flu-TCNQ. The good electron mobility is mainly attributed to the super-exchange effect in the Flu-TCNQ, where the D–A–D–A cluster provides effective channels for charge transport [47–49]. In our experiment, all the measurements were conducted under the ambient condition, indicating the good air stability of the crystal transistor. The high charge-carrier mobility property endows the Flu-TCNQ with potential application in advanced optoelectronic devices.

## Conclusion

A Flu-TCNQ cocrystal was prepared with a mixed packing mode and a 1:1 stoichiometric ratio of donor and acceptor. Driven by the strong CT interaction, the Flu-TCNQ exhibits integrated deep red emission and good electron mobility of 0.32 cm^2^ V^−1^ s^−1^, which can be considered as a candidate for integrated optoelectronic application. This work is significant for cocrystal engineering and provides guidance in designing and constructing advanced optoelectronic materials. However, there are still difficulties in balancing the optical and electrical properties of the cocrystals. More in-depth and detailed work is still in progress in our laboratory.

## Supplementary Information

**Additional file 1. Fig. S1.** Distance between donor and acceptor molecules was calculated by (*L*_donor_: the distance between two adjacent donor molecules; *L*_acceptor_: the distance between two adjacent accepotors molecules). **Fig. S2.** Intermolecular potential energy of Flu-TCNQ cocrystal. **Fig. S3.** TGA measurements of Flu, TCNQ, Flu-TCNQ. **Fig. S4.** Raman spectra of Flu, TCNQ, Flu-TCNQ. **Fig. S5.** Fluorescence microscopy image of Flu-TCNQ microwires. **Table S1.** Single crystal structure of Flu-TCNQ cocrystal.
