# Supplementary material for: TCNQ-based organic cocrystal integrated red emission and n-type charge transport
Source: Front Optoelectron. 2022 May 9;15(1):21. doi: 10.1007/s12200-022-00022-7 (PMC9756251; doi:10.1007/s12200-022-00022-7)
Supplement: Supplementary file 1 — Additional file 1. Fig. S1. Distance between donor and acceptor molecules was calculated by (Ldonor: the distance between two adjacent donor molecules; Lacceptor: the distance between two adjacent accepotors molecules). Fig. S2. Intermolecular potential energy of Flu-TCNQ cocrystal. Fig. S3. TGA measurements of Flu, TCNQ, Flu-TCNQ. Fig. S4. Raman spectra of Flu, TCNQ, Flu-TCNQ. Fig. S5. Fluorescence microscopy image of Flu-TCNQ microwires. Table S1. Single crystal structure of Flu-TCNQ cocrystal. [file 12200_2022_22_MOESM1_ESM.docx]

**Supporting Information**

**TCNQ-based organic cocrystal integrated red emission**

**and n-type charge transport**

Mengjia JIANG^1^, Shuyu LI^2^, Chun ZHEN^1^, Lingsong WANG^1^, Fei LI^1^, Yihan ZHANG^1^, Weibing Dong^3^,

Xiaotao ZHANG (✉)^2,3^, Wenping HU (✉)^1,4^

1 Tianjin Key Laboratory of Molecular Optoelectronic Science, Department of Chemistry, School of Science, Tianjin University, Tianjin 300072, China

2 Institute of Molecular Aggregation Science, Tianjin University, Tianjin 300072, China

3 Key Laboratory of Resource Chemistry and Eco-environmental Protection in Qinghai-Tibet Plateau, School of Chemistry and Chemical Engineering, Qinghai Minzu University, Xining 810007, China

4 Joint School of National University of Singapore and Tianjin University, International Campus of Tianjin University, Fuzhou 350207, China

Corresponding Authors: zhangxt@tju.edu.cn; huwp@tju.edu.cn


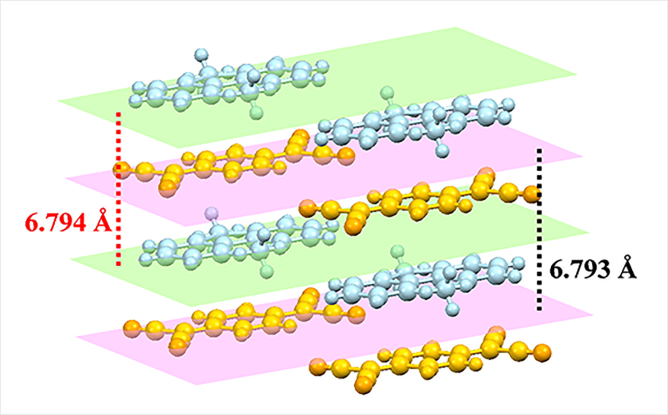


**Fig. S1** Distance between donor and acceptor molecules was calculated by $l=\frac{\text{L}_{\text{donor}}+\text{L}_{\text{acceptor}}}{4}$ (*L*_donor_: the distance between two adjacent donor molecules; *L*_acceptor_: the distance between two adjacent accepotors molecules.).


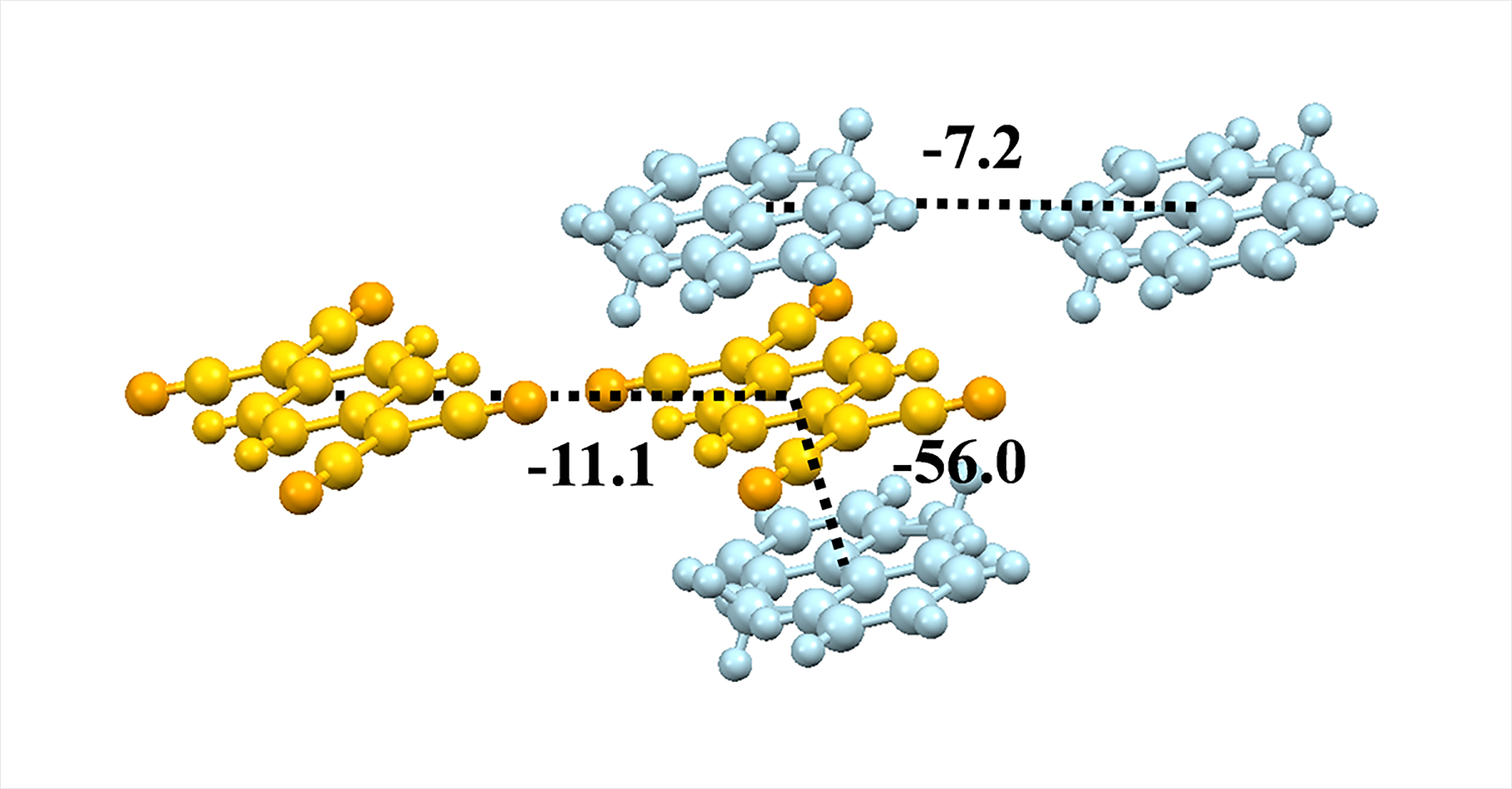


**Fig. S2** Intermolecular potential energy of Flu-TCNQ cocrystal.


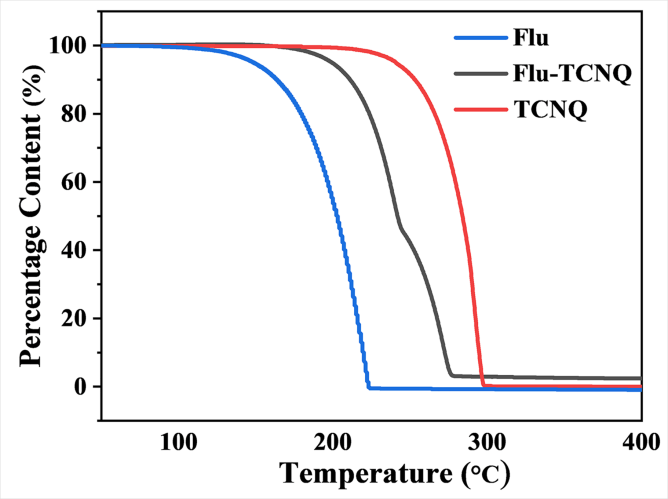


**Fig. S3** TGA measurements of Flu, TCNQ, Flu-TCNQ.


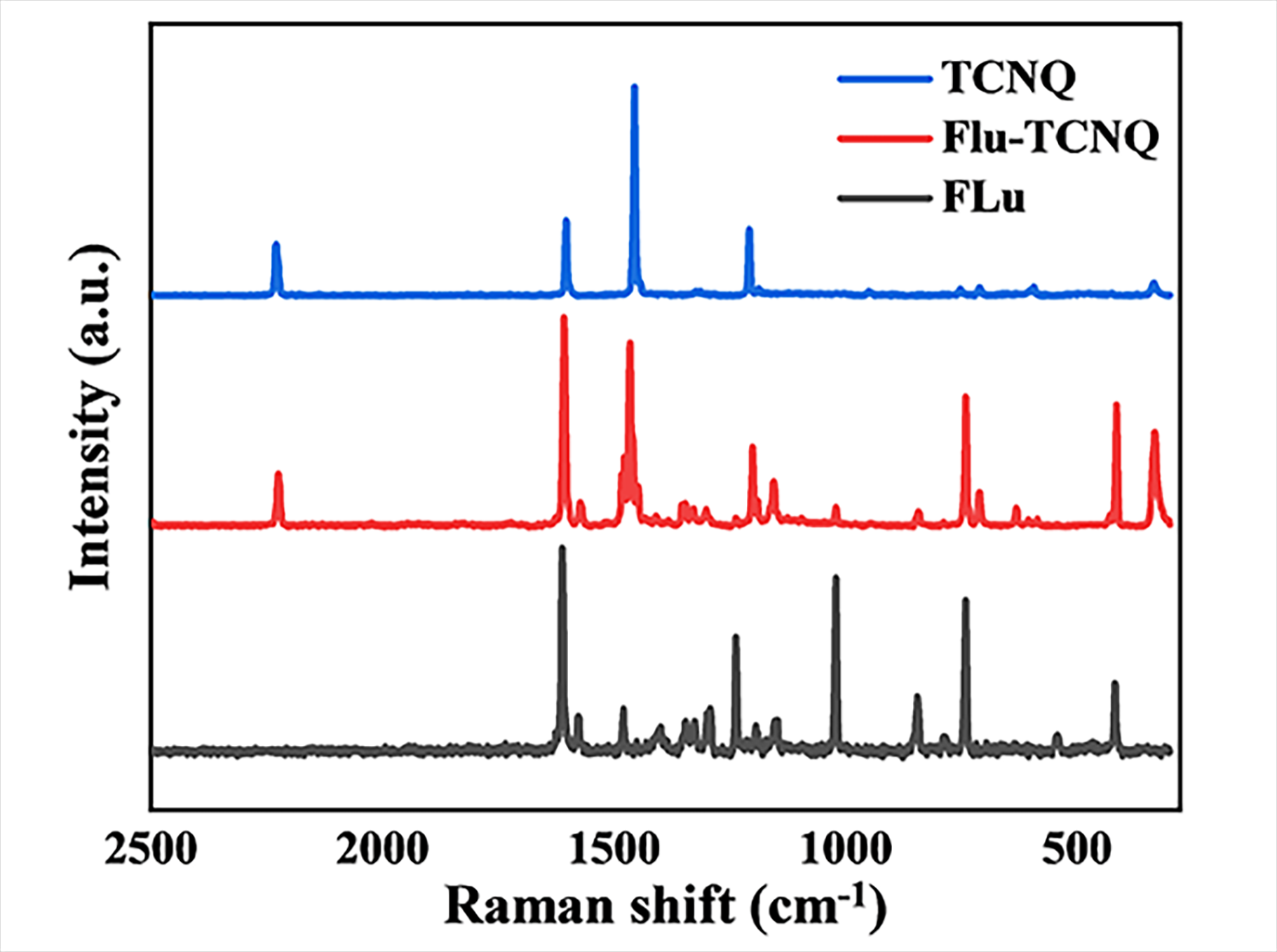


**Fig. S4** Raman spectra of Flu, TCNQ, Flu-TCNQ.


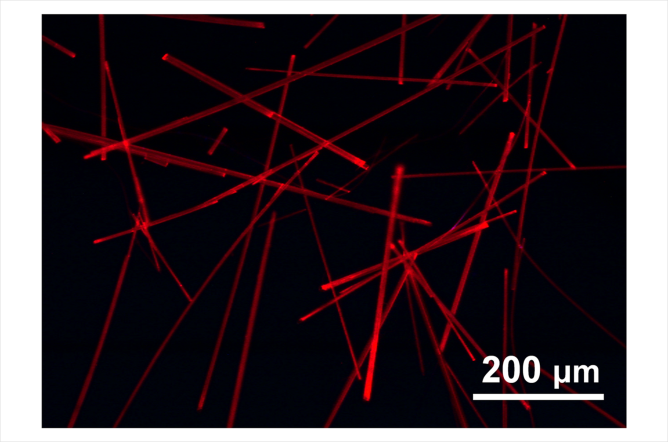


**Fig. S5** Fluorescence microscopy image of Flu-TCNQ microwires.

**Table S1** Single crystal structure of Flu-TCNQ cocrystal.

| **Crystal** | **Flu-TCNQ** |
| --- | --- |
| Empirical formula | C_25_H_14_N_4_ |
| Formula weight | 370.40 |
| Temperature/K | 300.15 |
| Crystal system | Monoclinic |
| Space group | C2/m |
| *a*/Å | 11.0097 (6) |
| *b*/Å | 13.1035 (6) |
| *c*/Å | 6.7937 (3) |
| *α*/(° ) | 90 |
| *β*/(° ) | 103.545 (5) |
| *γ*/(° ) | 90 |
| Volume/Å^3^ | 952.84 (8) |
| *Z* | 2 |
| *ρ*_calcg_/cm^3^ | 1.291 |
| *μ*/mm^-1^ | 0.619 |
| F (000) | 384.0 |
| Crystal size/mm^3^ | 0.12 × 0.11 × 0.10 |
| Reflections collected | 4572 |
| Independent reflections | 1019 [*R*_int_ = 0.0250, *R*_sigma_ = 0.0166] |
| Goodness-of-fit on *F*^2^ | 1.127 |
| Final *R* indices [*I*>2sigma(*I*)] | *R*_1_ = 0.0575, *wR*_2_ = 0.1777 |
| *R* indices (all data) | *R*_1_ = 0.0641, *wR*_2_ = 0.1892 |
